# Oscillatory motor network activity during rest and movement: an fNIRS study

**DOI:** 10.3389/fnsys.2014.00013

**Published:** 2014-02-04

**Authors:** Sahil Bajaj, Daniel Drake, Andrew J. Butler, Mukesh Dhamala

**Affiliations:** ^1^Department of Physics and Astronomy, Georgia State UniversityAtlanta, GA, USA; ^2^Department of Physical Therapy, Byrdine F. Lewis School of Nursing and Health Professions, Georgia State UniversityAtlanta, GA, USA; ^3^Department of Veteran's Affairs, Atlanta Rehabilitation Research and Development Center of ExcellenceDecatur, GA, USA; ^4^Joint Center for Advanced Brain Imaging, Center for Behavioral Neuroscience, Neuroscience Institute, Georgia State UniversityAtlanta, GA, USA

**Keywords:** fNIR, slow oscillations, resting state, motor networks, Granger causality, brain connectivity

## Abstract

Coherent network oscillations (<0.1 Hz) linking distributed brain regions are commonly observed in the brain during both rest and task conditions. What oscillatory network exists and how network oscillations change in connectivity strength, frequency and direction when going from rest to explicit task are topics of recent inquiry. Here, we study network oscillations within the sensorimotor regions of able-bodied individuals using hemodynamic activity as measured by functional near-infrared spectroscopy (fNIRS). Using spectral interdependency methods, we examined how the supplementary motor area (SMA), the left premotor cortex (LPMC) and the left primary motor cortex (LM1) are bound as a network during extended resting state (RS) and between-tasks resting state (btRS), and how the activity of the network changes as participants execute left, right, and bilateral hand (LH, RH, and BH) finger movements. We found: (i) power, coherence and Granger causality (GC) spectra had significant peaks within the frequency band (0.01–0.04 Hz) during RS whereas the peaks shifted to a bit higher frequency range (0.04–0.08 Hz) during btRS and finger movement tasks, (ii) there was significant bidirectional connectivity between all the nodes during RS and unidirectional connectivity from the LM1 to SMA and LM1 to LPMC during btRS, and (iii) the connections from SMA to LM1 and from LPMC to LM1 were significantly modulated in LH, RH, and BH finger movements relative to btRS. The unidirectional connectivity from SMA to LM1 just before the actual task changed to the bidirectional connectivity during LH and BH finger movement. The uni-directionality could be associated with movement suppression and the bi-directionality with preparation, sensorimotor update and controlled execution. These results underscore that fNIRS is an effective tool for monitoring spectral signatures of brain activity, which may serve as an important precursor before monitoring the recovery progress following brain injury.

## Introduction

Based on converging electrophysiological and neuroimaging data, the brain is known to be a self-organizing dynamical system consisting of anatomically distinct and efficiently connected brain regions supporting inherent electrical, chemical, hemodynamic, and metabolic processes (Buzsaki, 2006; Palva and Palva, 2012). An aspect of the brain's self-organizing dynamic behaviors is reflected in slow (<0.1 Hz) fluctuations (oscillations) of blood-oxygenation-level dependent (BOLD) functional magnetic resonance imaging (fMRI) signals recorded during resting conditions (Biswal et al., 1995; Bajaj et al., 2013). These intrinsic BOLD fluctuations are believed to be associated with neural level excitability fluctuations in cortical and subcortical networks (Buzsáki and Draguhn, 2004; Balduzzi et al., 2008; Keilholz et al., 2010) providing neural substrates for the flexibility and variability in moment-to-moment perception, cognition and motor behaviors (Arieli et al., 1996; Makeig et al., 2004; Palva and Palva, 2012). These slow coherent oscillations are the backbone of whole-brain functional connectivity networks such as default-mode networks (Raichle et al., 2001; Buckner et al., 2008), which have been intensely studied in basic and clinical neuroscience (Fox and Greicius, 2010; Gillebert and Mantini, 2013). Despite tremendous progress in understanding the intrinsic brain functional connectivity patterns, the underlying network oscillations (“roots of these patterns”) and modulations by task conditions have not been understood very well. In particular, the details on what oscillatory networks are at work during resting conditions and how these networks change in connectivity strength, frequency and direction when going from rest to explicit task are still being revealed. Here, we studied low-frequency network oscillations within the sensorimotor regions of able-bodied individuals during rest and motor movement using the hemodynamic activity as measured by functional near-infrared spectroscopy (fNIRS).

fNIRS technology uses specific wavelengths of light in the near-infrared range between 700 and 1000 nm, irradiated through the scalp, to enable the non-invasive measurement of changes in the relative ratios of deoxygenated hemoglobin (deoxy-Hb) and oxygenated hemoglobin (oxy-Hb) following neuronal activity in the brain (Arno and Britton, 1997; Kober et al., 2014). Like the BOLD fMRI, fNIRS measurements also reflect hemodynamic changes and rely on the neurovascular coupling in the brain (Ogawa et al., 1990; Logothetis et al., 2001; Raichle et al., 2001; Logothetis and Wandell, 2004) to infer changes in neural activity. Low frequency fluctuations have been observed in the fNIRS recordings of brain activity (Obrig et al., 2000; Duff et al., 2008). Obrig et al. (2000) have studied spectral power of fluctuations in fNIRS recordings and reported three spectral peaks around 0.03, 0.1, and 1 Hz during resting conditions and during visual stimulation tasks. There was an increase in oxy-Hb concentration and a decrease in deoxy-Hb during visual stimulation. Amplitude modulations of low-frequency oscillations (LFO) have been observed in task-activated and task-deactivated regions (Duff et al., 2008). However, there have not been fNIRS studies to provide evidence of brain intrinsic network oscillations and their modulations by task conditions.

Previous studies have focused on brain functional and effective connectivity during resting state (RS), motor imagery (MI), and motor execution (ME) tasks using fMRI and EEG. Most of these studies compare the connection strengths and directionality between MI and ME tasks and demonstrate modulation during task execution (Solodkin et al., 2004; Grefkes et al., 2008; Kasess et al., 2008; Chen et al., 2009; Gao et al., 2011). These studies suggest that the networks during MI and ME behave in a similar way or at least have some common and overlapping networks involving the brain areas including the primary motor cortex (M1), the premotor cortex (PMC) and the supplementary motor area (SMA) (Jeannerod, 1994; Gerardin et al., 2000; Solodkin et al., 2004; Kasess et al., 2008). These studies are based on independent hypotheses and estimations are performed using statistical models, which are further based on anatomical assumptions reflecting basic structural connections among these cortical areas. DCM (Friston et al., 2003) and Granger causality (GC) (Granger, 1969; Friston et al., 2003) are the two most common computational approaches used to analyze effective connectivity or directed functional connectivity between brain regions. It has been found that during ME there is time lag in activation pattern between M1 and SMA suggesting a delayed response of M1 during task execution (Kasess et al., 2008). Anatomically, SMA is reciprocally connected to M1 causing bilateral activity even for unimanual hand movements (Muakkassa and Strick, 1979; Deecke, 1987). A comparison of connectivity between RS and ME using the DCM approach suggests that parameters for RS and condition-specific DCM connectivity parameters during motor task are weakly correlated, whereas task-based parameters are strongly and positively correlated with DCM connectivity parameters (Rehme et al., 2013). Jiang et al. (2004) showed that a functional connectivity in the motor networks during rest can be modulated by planning, initiation and coordination of voluntary movements.

Reports of network activity in unilateral and bilateral hand (BH) movements have found that during right hand movements, left SMA and left premotor cortex (LPMC) promote activity in left primary motor cortex (LM1) positively whereas networks are modulated negatively toward right M1 (Grefkes et al., 2008). Conversely during bimanual hand movements, both right and left M1 are positively modulated. Others have shown that activity during bimanual movement is initiated by the dominant hemisphere (Walsh et al., 2008). DCM results suggest that during ME task there is weak positive influence from SMA on M1 whereas during kinesthetic MI, the influence is suppressive (Solodkin et al., 2004; Kasess et al., 2008). Their study also suggests that the feedback connection from M1 to SMA may play a significant role in the preparation and coordination of a task. Previous studies also confirmed that connectivity parameters of motor networks could change in stroke survivors. This was demonstrated by larger individual path variance in patients in comparison to healthy subjects confirmed by the diminishing connections for M1 and SMA (Inman et al., 2012). It is still not clear how (at what frequency, for example) M1 and SMA exert influence on each other during ME and what role this feed-forward or feed-backward circuit plays in initiating and executing motor tasks.

In the current study, we used near-infrared spectroscopy (NIRS), a relatively new technique as compared to other neuroimaging modalities, to investigate the directed functional connectivity using GC approach during rest as well as during ME. NIRS can only be used to measure activities on cortical surfaces whereas fMRI can be used to measure activations throughout the whole brain. However, NIRS is a non-invasive, safe, cost effective and more flexible and portable technique with reasonable spatial and excellent temporal resolution compared to fMRI. NIRS also allows monitoring children as well as patients who are psychologically unfit to be studied under traditional neuroimaging methods.

Considering the above-mentioned advantages of NIRS over fMRI, our present study explores and compares the cortical network dynamics during RS, between task resting state (btRS) and ME tasks using the parametric GC approach (Granger, 1969; Dhamala et al., 2008b) focusing on the motor network consisting of: primary motor cortex (M1), PMC and the SMA. The primary goals of the current study were to: (i) evaluate the intrinsic oscillatory features at low frequencies (0.01–0.1 Hz) and the task modulations and (ii) establish the characteristics of normal brain motor network activity as a necessary precursor prior to the study of network activity following brain injury. A secondary goal was to assess the applicability of NIRS as a tool to explore the functional connectivity among different brain areas. Most of the previous fNIRS studies investigate functional connectivity among various brain areas during much simpler hand motor tasks. The question remains is to whether NIRS can be used effectively to infer how neural activations respond to various complex motor tasks.

## Materials and methods

### Participants

Twenty-seven able-bodied adult volunteers participated in the study (8 males; 19 females; age 22–63, mean = 31.8 ± 12.8 years). People were excluded from the study if they: (a) had any medical conditions that could interfere with ability to complete questionnaires and visual-motor tasks, (b) had any unstable medical conditions, (c) were unable to attend both testing sessions, or (d) were taking any medications which could influence motor or visual ability. All subjects provided written informed consent and procedures were reviewed and approved by the local Institutional Review Board (IRB).

### NIRS recordings

During recordings, participants were seated comfortably in an adjustable chair facing a computer screen approximately 24 inches away. Data was collected in two 1-h sessions separated by 7 days using the Hitachi ETG-4000 52-channel NIRS system (Hitachi Medical Co., Tokyo Japan). The absorption of near infrared light at two wavelengths (695 and 830 nm) was measured with a sampling rate of 10 Hz. Changes in reduced (deoxy) hemoglobin (HbR), and oxy-Hb (HbO) concentrations at each time point from each channel were computed using the modified Beer-Lambert law (Cope et al., 1988). The 8 emitter probes and 7 detector probes were arranged into an 3× 5 array, resulting in 22 measurement channels (inter-optode distance = 30 mm).

### Cap positioning and measurement

**Figure 1:**
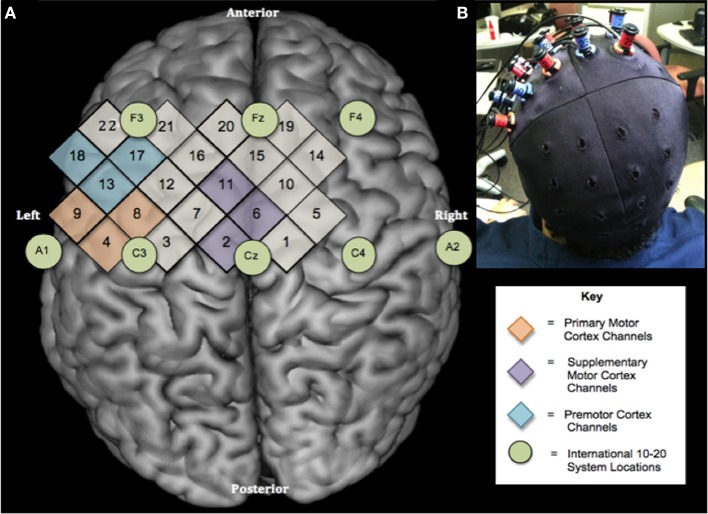
**Experimental design. (A)** Location of NIRS channels relative to the International 10–20 System shown on a standard brain template (Rorden et al., 2007). Numbers in diamonds indicate individual NIRS channels. Circles represent locations on the International 10–20 System. There are 2 cm between the centers of neighboring channels **(B)** Optode configuration and cap placement for sessions 1 and 2.

During testing, cap position was adjusted to make sure that the hair bundles were not blocking sensors and detectors, a strong evoked activity occurred over sensorimotor regions for ME and adequate signals were obtained from all the channels (Figure 1A). The cap was made of an elastic spandex-type fabric in which the grid was embedded (Figure 1B). Standard cap measurements for each subject were then taken relative to prominent anatomical landmarks. In order to replicate the cap position across sessions, the following distances were measured: the distance (in mm) from the left and right tragus to the center point of the cap, nasion to cross seam, and inion to center point.

The position of the cap and ROIs were determined by calculating *x* and *y* distance between the center of the cap and all optode and channel locations. The Cz location, defined as the intersection point of two lines formed by 50% of the distance between the left and right tragus and 50% of the distance between the nasion and inion (Jasper, 1958), was calculated for each subject individually based on anatomical measurements obtained during data collection. Then, the coordinates(*x, y*) of the cap seam were subtracted from the Cz locations for each subject in order to calculate the distance between the subject's true Cz and the cap cross hair/center. The position of the cap seam relative to anatomical landmarks was measured for every participant at each session. Last, the coordinates (*x, y*) of the optodes and channels were calculated by subtracting the amount of cap shift (true Cz—cap seam) from each individual optode and channel position (*x, y*) on the cap. Thus, the exact location of each optode was calculated for subjects individually.

### Protocol

During each fNIRS recording session, participants underwent a 7 min RS followed by a 9 min 30 s motor task. Each participant read a standardized set of task instructions before each recording to ensure normalization of procedures within and between sessions. The RS occurred prior to the motor task for all participants at each session in order to control for possible confounding effects of task performance (Fransson, 2006). During the RS measurement, subjects were asked to refrain from cognitive, motor and language tasks while visualizing a fixation-cross (Greicius et al., 2003). The motor task involved performing a finger tapping sequence with hands resting palms down (Verstynen et al., 2005). Participants performed three cycles of the following sequence: right-hand (RH), left-hand (LH), and BH finger tapping. Each tapping interval lasted 30 s. Thirty-second rest intervals were included between tapping segments in which subjects visualized a fixation cross and remained motionless. The bilateral finger-tapping task was included to facilitate activity in the SMA, which has been shown to demonstrate increased activation during simultaneous right and left finger movements (Debaere et al., 2001).

### ROI selection

Three ROIs were defined anatomically as follows: the M1 as the area extending from the anterior bank of the central sulcus to the anterior edge of the precentral gyrus (Dassonville et al., 2001; Kimberley et al., 2008b,c), the PMC as the area between M1 and the sulcus nearest the coronal plane through the anterior commissure, bounded inferiorly by the inferior edge of the frontal lobe (Dassonville et al., 2001), and the SMA as the medial region of the hemispheres superior to the dorsal bank of the cingulated sulcus along the same anterior-posterior extent as the PM (Dassonville et al., 2001; Kimberley et al., 2008a,c).

Each ROI was assigned a position within the international 10–20-electrode system, which corresponded to its anatomical location based on previous research and neuroanatomical atlases (Jasper, 1958; Homan et al., 1987). Based on the 10–20 international system, M1 ROI was assigned to the C3, PMC ROI as 50% of the distance between Cz and F3, and SMA ROI as 50% of the distance between Cz and Fz. The coordinates of the selected international 10–20 system positions were calculated for each subject.

For channel selection, the three closest channels to the international 10–20 system locations were calculated mathematically for each participant and selected to represent each ROI. The channels closest to ROIs were the same for every subject for M1 (channels 4, 8, and 9), SMA (channels 2, 6, and 11), and PM (channels 13, 17, and 18) (Figure 1A).

### Data preprocessing and analysis

Raw fNIRS data were linearly detrended, band-pass filtered between 0.01 and 0.1 Hz. The detrending and filtering removed slow trends and other physiological noise such as respiration and cardiac activities.

In the resting condition, participants visualized a fixation cross and the recordings form the baseline (extended RS). The second condition was the task condition, which had fNIRS data for: (i) resting period prior to each task period, i.e., btRS and (ii) during task periods, i.e., during ME. During ME, time series of whole length (9 min 30 s) from each node and run was broken into segments each of length 30 sec. These segments were grouped together separately for different conditions: btRS, RH, LH, and BH finger tapping. Each movement condition was repeated three times and rest condition occurred for a total of 10 times. These repetitions were treated as trials for the spectral analysis. For each condition, concentration changes for oxy-Hb, deoxy-HB, and total-Hb were calculated. We used only oxy-Hb signal changes in current analysis since previous studies showed no significant correlation between changes in deoxy-Hb and ability to imagine movements measured by MIQ-R (Kober et al., 2014), whereas under task-related activations, oxy-Hb signal was more robust and highly stable with time than deoxy-Hb signals (Obrig et al., 2000; Mihara et al., 2012). The ensemble means of the segments for each node (M1, SMA, PM) were removed from the segmented data to make these zero-mean processes for spectral analysis. We then computed power, coherence and GC using the parametric spectral approach (Dhamala et al., 2008b). Coherence and GC are categorized as spectral interdependency measures, which are used to characterize network oscillations.

### Spectral interdependency measures

Spectral interdependency measures are a means of statistically quantifying the inter-relationship between a pair of oscillatory processes; say 1 and 2, as a function of frequency of oscillation. In practice, there are three measures that characterize the spectral interdependency between a pair of processes: total interdependence (*M*_1, 2_), GC one-way effect or directional influence from the first process to the second process (*M*_1 → 2_) or from the second to the first (*M*_2 → 1_) and instantaneous causality (measure of reciprocity, *M*_1.2_). These are related as (Geweke, 1982):

(1) $$M_{1,\;2}=M_{1\to 2}+M_{2\to 1}+M_{1.2}$$

The measures of spectral interdependency are derived from a spectral density matrix (S), which is constructed from the time series of oscillatory systems by using optimal autoregressive (AR) modeling in the parametric method.

Diagonal elements of the spectral density matrix (S) represented node activity in terms of spectral power (P), whereas the coherence function *C*(*f*) was derived from the cross spectra normalized by the product of the individual auto spectra as:

(2) $$C\left(f\right)=\frac{|S_{12}\left(f\right){|}^{2}}{S_{11}\left(f\right)S_{22}\left(f\right)}$$

The coherence function is a well-accepted measure to characterize frequency-specific interdependence between multiple time series from multisite recordings such as multi-electrode electrophysiological recordings, electro/magneto encephalography (E/MEG) and functional magnetic resonance imaging (fMRI). It ranges from 0 (no interdependence) to 1 (maximum interdependence). *C*(*f*) is related to Geweke's measure of total interdependence (*M*_1, 2_) (Geweke, 1982):

(3) $$M_{1,\;2}\left(f\right)=-\ln\text{​}\left(1-C\left(f\right)\right)$$

whose value ranges from 0 to infinity.

Directional influences between processes 1 and 2 are given by (Geweke, 1982; Ding et al., 2006; Dhamala et al., 2008a,b):

(4) $$\begin{array}{l} M_{1\to 2}\left(f\right)=\text{ln}\frac{S_{22}\left(f\right)}{{\tilde{H}}_{11}\left(f\right)\Sigma_{11}{\tilde{H}}_{11}^{*}\left(f\right)}\\ M_{2\to 1}\left(f\right)=\text{ln}\frac{S_{11}\left(f\right)}{{\tilde{H}}_{22}\left(f\right)\Sigma_{22}{\tilde{H}}_{22}^{*}\left(f\right)}, \end{array}$$

where ${\tilde{H}}_{11}=H_{11}+\frac{\Sigma_{12}}{\Sigma_{11}}H_{12}$, ${\tilde{H}}_{22}=H_{22}+\frac{\Sigma_{12}}{\Sigma_{22}}H_{21}$

Here, ∑ (noise covariance matrix), H (transfer function matrix), $\tilde{H}$(new transfer function matrix) are estimated from the residual errors and the inverse of the Fourier transforms of the coefficients in AR models respectively. Here ^*^ denotes matrix adjoint.

### Significant tests and percentage modulations

GC values were integrated over the frequency range from 0.01 Hz (*f*_1_) to 0.1 Hz (*f*_2_):

(5) $$iGC_{1\to 2}=\frac{1}{f_{2}-f_{1}}\int_{f_{1}}^{f_{2}}M_{1\to 2}(f)df$$

Significant connections for each condition (RS, btRS, RH, LH and BH finger tapping) were found using permutation test at *p* < 0.05. Further, a two-sample *t*-test for all the significant connections was performed for ME task (LH, RH and BH finger tapping) vs. RS. We considered RS and btRS conditions as reference conditions- reference 1 (ref 1) and reference 2 (ref 2) respectively. The percentage of modulation between two nodes, for example 1 and 2, having significant connection strength for a particular condition (LH, RH, BH finger tapping) relative to the reference conditions were calculated as follows:

(6) $$M=\frac{iGC_{ME}-iGC_{ref1\left(or2\right)}}{iGC_{ref1\left(or2\right)}}\times 100\%$$

where *M, iGC*_*ME*_ and *iGC*_*ref1(or2)*_ represented percentage modulation, integrated causal flow for ME and integrated causal flow for ref 1 (or 2) from node 1 to node 2 respectively.

## Results

### Power, coherence, and GC spectra

**Figure 2:**
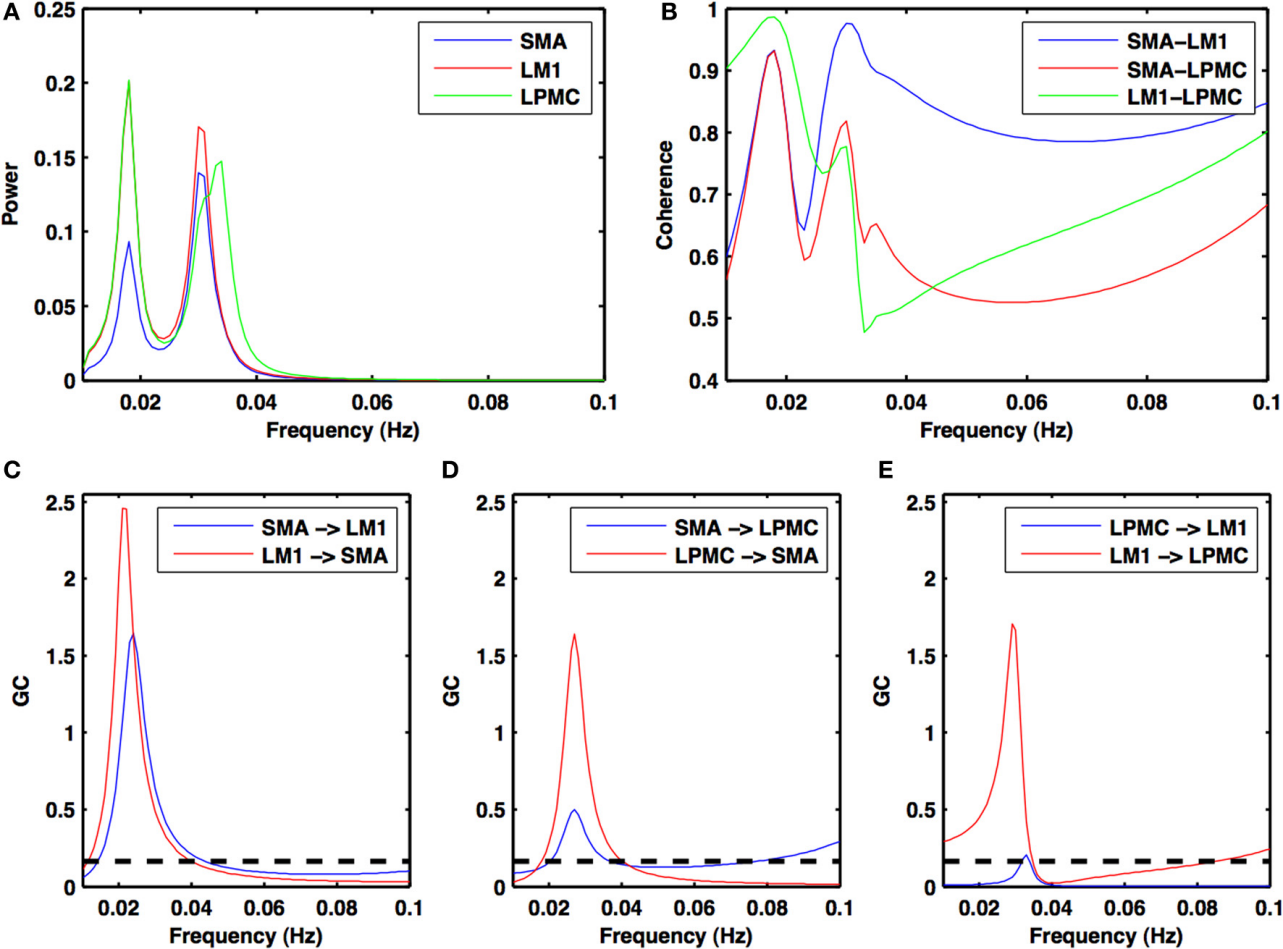
**Power, coherence and Granger causality spectra during resting state**. For all the three nodes **(A)** peaks for power spectra for SMA (blue), LM1 (red), and LPMC (green) **(B)** high coherence values among all the combinations: SMA-LM1 (blue), SMA-LPMC (red) and LM1-LPMC (green) and peaks for GC for **(C)** SMA-LM1 (blue), LM1-SMA (red) **(D)** SMA-LPMC (blue), LPMC-SMA (red), and **(E)** LPMC-LM1 (blue), LM1-LPMC (red) are obtained in the same frequency band of 0.01–0.04 Hz. Dashed black lines in GC plots show significant threshold at *p* < 0.01 (*n* = 27).

**Figure 3:**
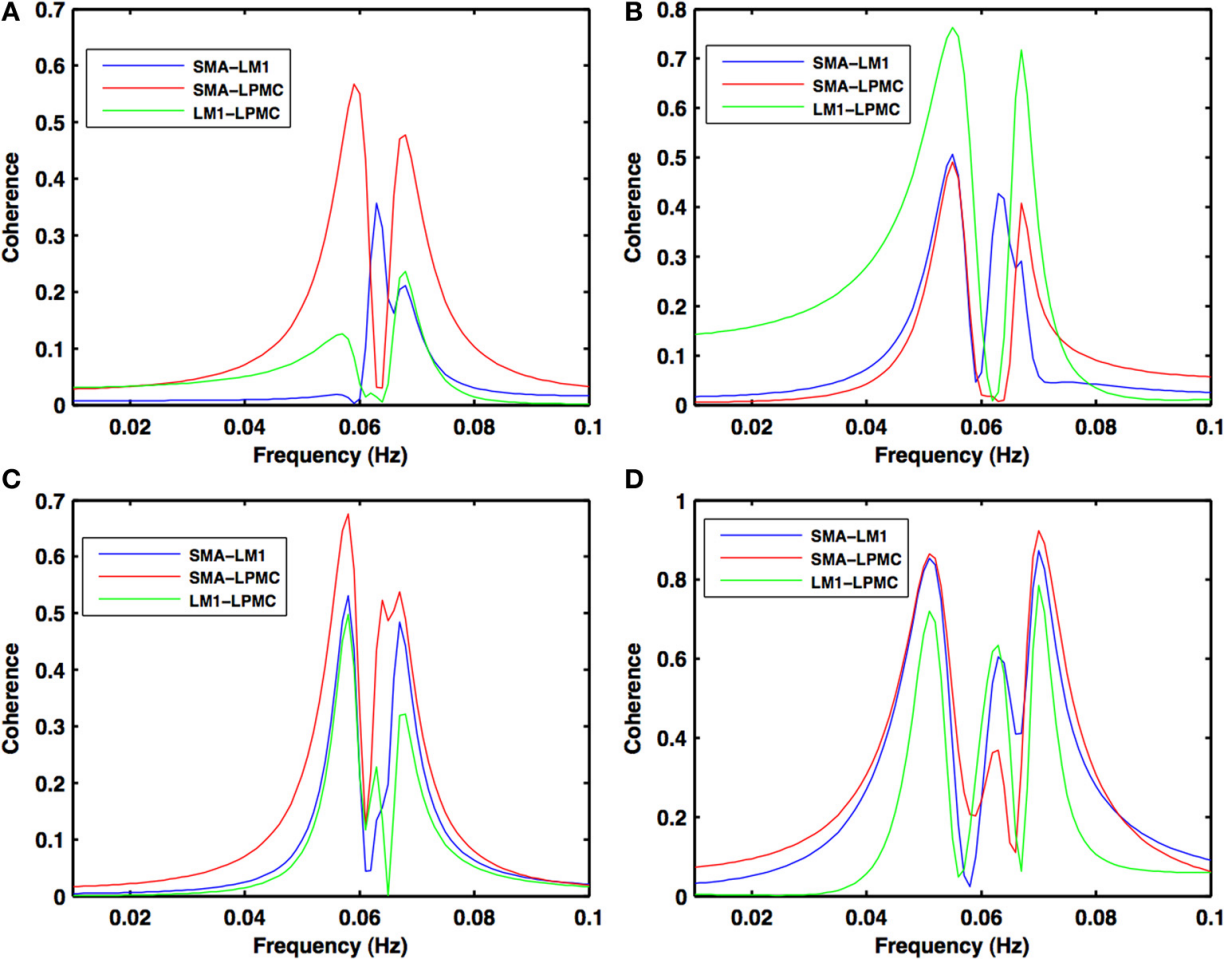
**Coherence spectra during motor execution**. High coherence values among all the combinations: SMA-LM1 (blue), SMA-LPMC (red), and LM1-LPMC (green) are obtained in the frequency band of 0.04–0.08 Hz for **(A)** btRS condition **(B)** RH **(C)** LH, and **(D)** BH finger movement.

**Figure 4:**
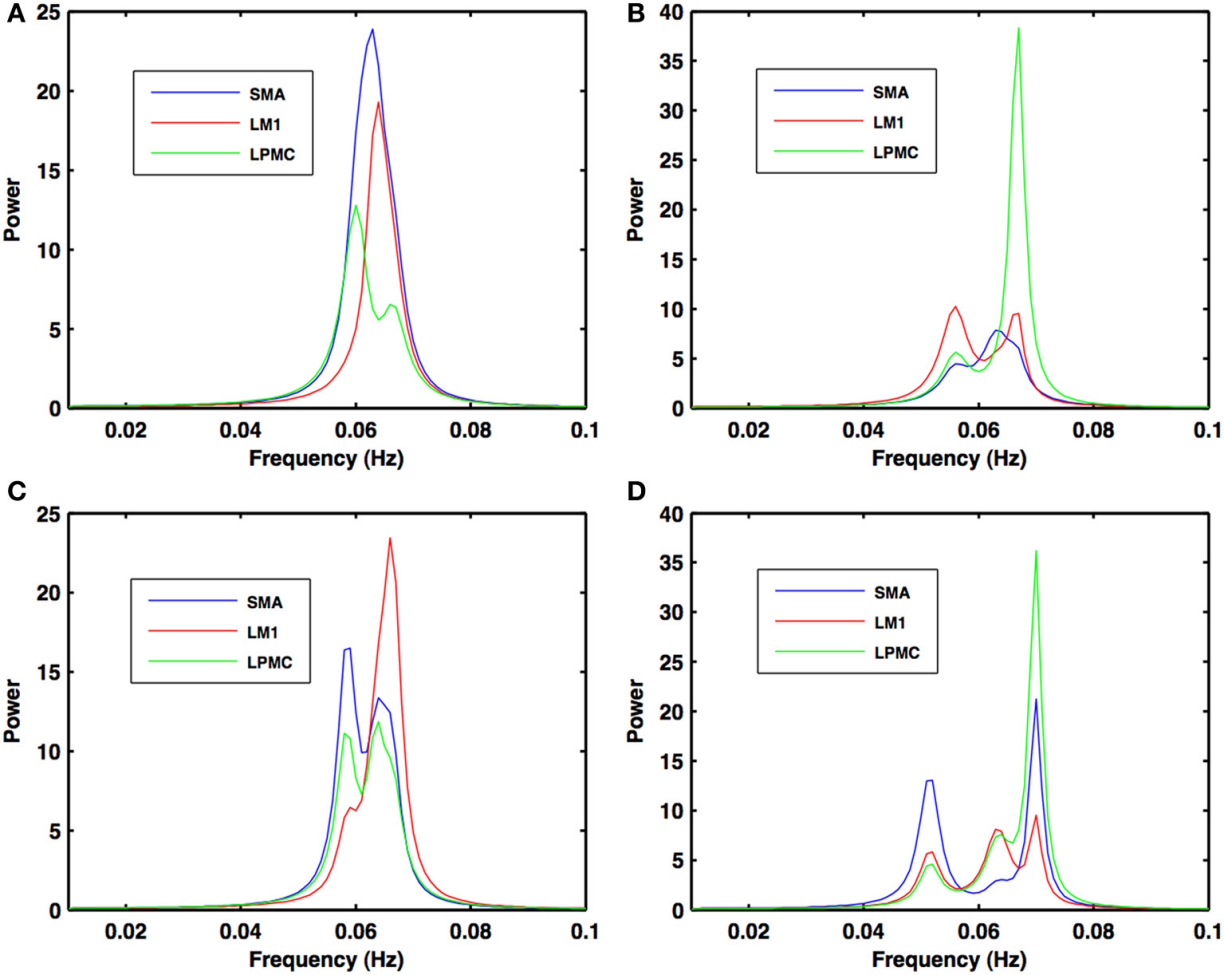
**Power spectra during motor execution**. Power peaks are obtained in the frequency band (0.04–0.08 Hz) for all the nodes: SMA (blue), LM1 (red), and LPMC (green) for **(A)** btRS condition **(B)** RH **(C)** LH, and **(D)** BH hand finger movement.

**Figure 5:**
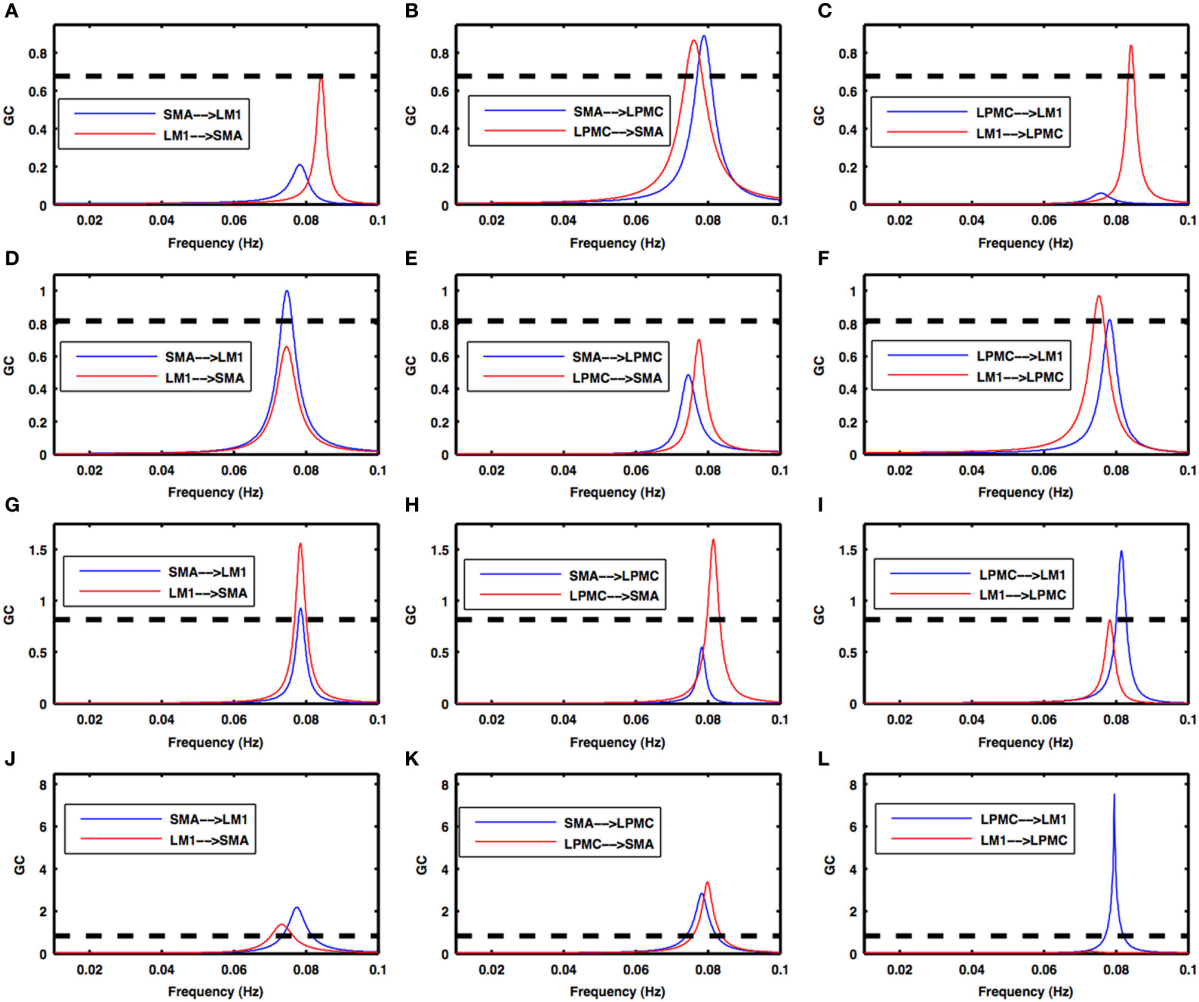
**Granger causality spectra during motor execution**. GC peaks for all the combinations among SMA, LM1, and LPMC are within 0.06–0.1 Hz for all the conditions- **(A–C)** btRS condition, **(D–F)** RH, **(G–I)** LH, and **(J–L)** BH. Dashed lines in these plots show a significant threshold (*p* < 0.01, *n* = 27).

Power, coherence and GC spectra for all the nodes (SMA, LM1, and LPMC), which were found to be involved during RS, btRS and task execution, were computed. Figures 2–5 show group level comparison of these spectra between two conditions: RS and motion execution (ME) by considering all the subject's runs as trials. Here within the ME condition, there are three sub-conditions: btRS, RH, LH, BH finger tapping.

During the RS, for all the three nodes, the peaks for power were in the frequency band 0.01–0.04 Hz (Figure 2A). All the nodes were found to be highly coherent (Figure 2B) in the same frequency band. Further GC peaks were also within same frequency band (0.01–0.04 Hz) (Figures 2C–E). On the other hand, for ME case, these nodes were highly coherent in the frequency band 0.04–0.08 Hz along with power peaks within same frequency band whereas GC peaks were within 0.06–0.1 Hz for all the conditions- btRS (Figures 3A, 4A, 5A–C, respectively), RH (Figures 3B, 4B, 5D–F, respectively), LH (Figures 3C, 4C, 5G–I, respectively), and BH (Figures 3D, 4D, 5J–L, respectively). Dashed lines in the GC plots show a significant threshold (*p* < 0.01, *n* = 27) (Figures 2C–E, 5).

### Directed functional connectivity

#### Condition 1: resting state (RS)

**Figure 6:**
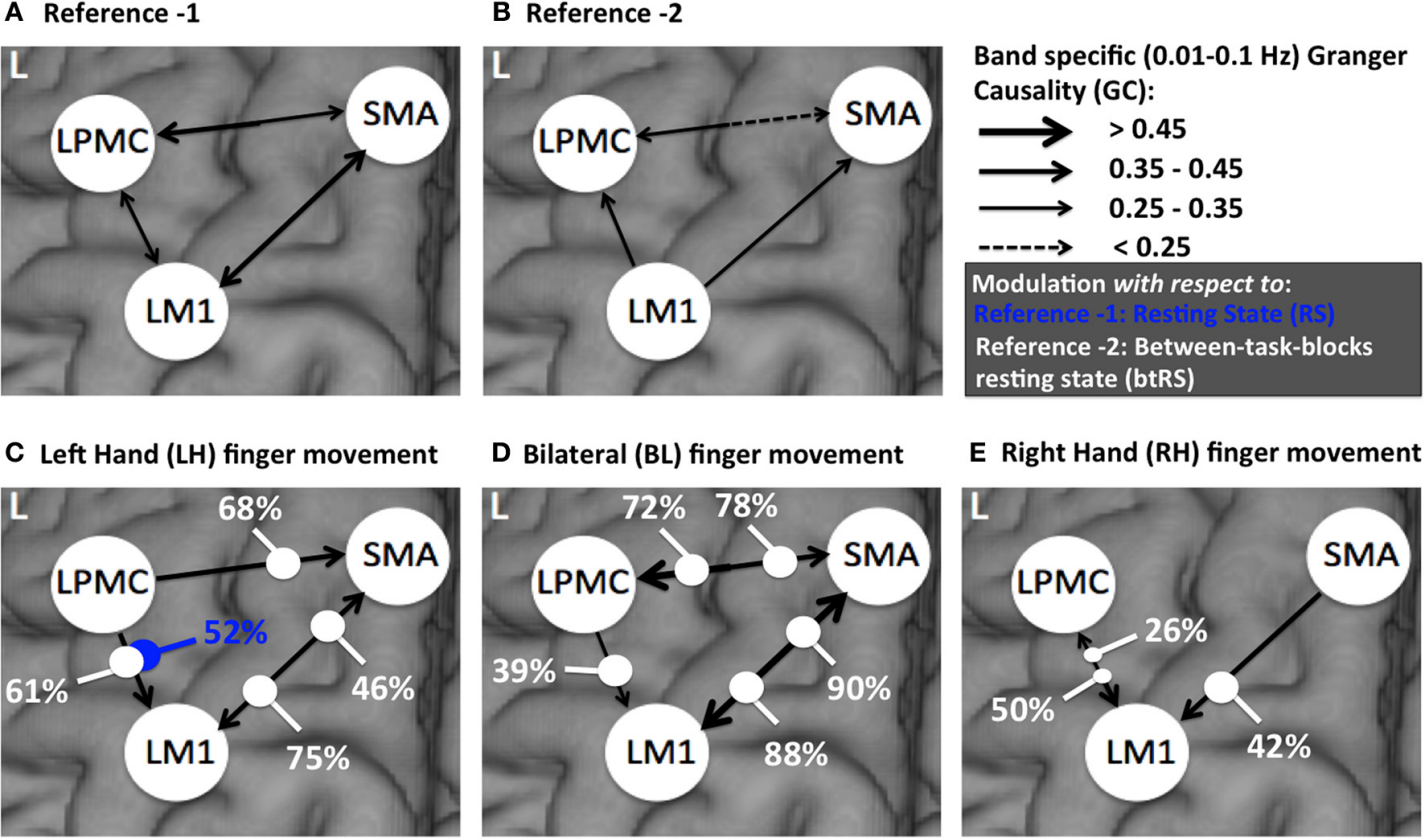
**A comparison of directed connectivity measures obtained by integrating over entire frequency band of interest (0.01–0.1 Hz) for **(A)** RS condition (reference 1) **(B)** btRS condition (reference 2) **(C)** LH **(D)** BH, and **(E)** RH finger movement**.

Directionality of causal flow among the three nodes, SMA, LM1, and LPMC was computed during RS. A bidirectional causal flow was observed among all the three nodes (Figures 2C–E). Dashed line in the plots shows the significant threshold (*p* < 0.01, *n* = 27). Although all the connections were bidirectional and significant there was considerable causal flow difference between forward and backward connections. In Figure 2C, causal flow from LM1 to SMA is more than SMA to LM1, in Figure 2D, causal flow from LPMC to SMA is more than SMA to LPMC and similarly in Figure 2E, causal flow is more from LM1 to LPMC than from LPMC to LM1. The overall calculation of causal flow was estimated by integrating over the entire frequency band (0.01–0.1 Hz) as shown in Figure 6A, reference 1). Here thickness of the arrows reflects magnitude of the causal flow.

#### Condition 2: motion execution (ME)

Similarly, directionality of causal flow among all the three nodes was computed and compared among btRS condition, RH, LH, and BH finger movement.

***Condition 2a***. *btRS condition-* Figures 5A–C shows the directionality of causal flow for btRS condition in ME task. A significant causal flow was found from LM1 to SMA (Figure 5A), bidirectional significant causal flow between SMA and LPMC (Figure 5B) and significant causal flow from LM1 to LPMC (Figure 5C).

***Condition 2b***. *RH finger movement-* Figures 5D–F shows the directionality of causal flow for RH finger movement condition in ME task. Here there was significant causal flow from SMA to LM1 (Figure 5D), insignificant causal flow between SMA and LPMC (Figure 5E) and bidirectional significant causal flow between LM1 and LPMC (Figure 5F).

***Condition 2c***. *LH finger movement-* Figures 5G–I shows the directionality of causal flow for LH finger movement condition in ME task. Here there was bidirectional significant causal flow between SMA and LM1 (Figure 5G), significant causal flow from LPMC to SMA (Figure 5H) and bidirectional significant causal flow between LM1 and LPMC (Figure 5I).

***Condition 2d***. *BH finger movement-* Figures 5J–L shows the directionality of causal flow for BH finger movement condition in ME task. Bidirectional significant causal flow was observed between SMA and LM1 (Figure 5J), bidirectional significant causal flow between SMA and LPMC (Figure 5K) and significant causal flow from LPMC to LM1 (Figure 5L).

#### A comparison: condition 1 vs. condition 2 and condition 2a vs. condition 2(b-d)

Considering RS condition (condition 1) as reference 1 (Figure 6A) and btRS (condition 2a) as reference 2 (Figure 6B), a pairwise *t*-test was performed for only significant connections in all the conditions i.e., reference 1 vs. reference 2 and RH, LH, and BH finger movement conditions 2(b-d) vs. reference 1 and reference 2. We found that none of the significant connections were significantly different for reference 1 vs. reference 2. There was only one connection, which was significantly different: LPMC to LM1 in reference 1 vs. LH condition (condition 2c), which is 52% modulated (Figure 6C, marked with blue dot).

For reference 2 vs. LH, we found all the connections were significantly different. There was modulation of 75% and 46% from SMA to LM1 and LM1 to SMA respectively. Further, there was modulation of 68% and 61% from LPMC to SMA and LPMC to LM1 respectively (Figure 6C).

For reference 2 vs. BH, all the connections were significantly different except from LPMC to LM1, which was, 39% modulated. There was 90 and 88% modulation from LM1 to SMA and SMA to LM1 respectively and 78 and 72% modulation from LPMC to SMA and SMA to LPMC respectively (Figure 6D).

For reference 2 vs. RH, there was only one connection SMA to LM1, which was significantly different and was 42% modulated. There was 50 and 26% modulation for significant connection from LPMC to LM1 and LM1 to LPMC respectively (Figure 6E).

## Discussion

We have observed differences in oscillatory motor network activity within the sensorimotor regions of able-bodied individuals during rest and finger movement using the hemodynamic activity as measured by fNIRS. During RS, there were significant node and network oscillations in the frequency band 0.01–0.04 Hz. These GC results obtained from the parametric approach showed that there were significant bidirectional connections among LM1, LPMC, and SMA during RS, from LM1 to SMA and LPMC and between SMA and LPMC during btRS. There were significant modulations between connections during ME task in comparison to btRS, especially, from SMA to LM1 during RH finger movement and bidirectional significant modulations between LM1 and SMA during LH and BH finger movements. We found significant positive modulations from LPMC to LM1 under all the conditions whereas LPMC to SMA during LH and bidirectional positive modulations between these during BH finger movement.

### Power, coherence, and GC spectra

Our findings confirm and extend previous findings that showed significant network activity changes in going from RS to movement (Jiang et al., 2004). Furthermore, strong coherence relationships in the frequency band 0.02–0.15 Hz are obtained between M1 and SMA (Otten et al., 2012). In the same study by Otten and colleagues, higher correlation values are obtained among motor areas in RS oscillations even in the presence of lesions. Further, basic motor networks are suggested to be spatially similar between patients and controls. Similarities have been found between anatomical and functional mapping using covariance analysis following RS MRI (Xiong et al., 1999). In animal studies, a spontaneous influence of stimulation on oxygenation and metabolism is found around 0.1 Hz (Mayhewa et al., 1999). It is suggested that these oscillations follow electrocorticography (EcOG) bursts of a specific type and can be evoked by a stimulus (Obrig et al., 2000).

Dominant components of LFO below 0.1 Hz is found using Fourier analysis of temporal components of oxy-hemoglobin signal in an NIRS study (Tong and Frederick, 2010). It has also been reported that frequency corresponding to task related activations had an amplitude around seven times greater than during RS activation maps (Cordes et al., 2001). Our study confirms and specifies these findings, suggesting a particular frequency band 0.01–0.04 Hz is dominant during RS and 0.04–0.08 Hz is dominant during btRS and ME tasks. Here we note that coherence spectra are related to the total interdependence (Equation 3) and the total interdependence is the sum of two one-way effects and instantaneous causality (Equation 1). The peak frequencies detected by coherence and GC methods thus may differ depending on the instantaneous causality. Dominant frequency bands of GC for RS and ME are different since for RS NIRS data analysis, there is no contrast used to explore the connections. Because brain areas are expected to be more coherent during RS than during ME task, RS is usually considered as baseline during analysis of task dependent networks. During btRS and ME tasks, integrated activity of several brain areas are necessary to perform a particular task. On the other hand, RS analysis is supposed to detect a complete but phase locked neural networks whereas task execution is supposed to detect all possible connections associated with a brain area. This suggests that there is some contribution by functional connectivity during RS to task related connectivity, both <0.1 Hz.

### Directed functional connectivity

#### (a) RS vs. btRS

Comparing the connection strengths and directionality between RS and btRS conditions, there are significant bidirectional connections between SMA and LPMC although weaker in case of btRS. Furthermore, there is unidirectional significant causal flow from LM1 to SMA during btRS in comparison to significant bidirectional causal flow between them in RS. Although, participants are not directly instructed to imagine motor task in between two ME tasks, our results are consistent with MI conditions where participants are usually instructed to imagine some hand or finger movements (Kasess et al., 2008). It has been shown that during MI, dorsal premotor (PMd) cortex have the primary influence on SMA and then backward from SMA to PMd (Matsumoto et al., 2007). Others have demonstrated suppressive influence of SMA on LM1 during MI, reporting a feedback circuit from LM1 to SMA (Kasess et al., 2008; Chen et al., 2009). The result could reflect the fact that SMA plays a crucial role in maintaining the activity within itself before passing information to neighboring areas in order to execute the motor plan. Although not demonstrated in the current study, negative values of LPMC on LM1 during MI (Solodkin et al., 2004) suggests the weak and suppressive influence of LPMC on LM1.

#### (b) Role of SMA, LPMC and LM1 during ME task

The connection from LPMC to LM1 was found to be significantly stronger in LH finger tapping in comparison to RS. This connection is also significantly stronger in all three conditions (LH, BH, and RH) when compared to btRS. During execution of task, whether unimanual or bimanual, both SMA and LPMC were shown to have a significant influence on LM1. The extent of modulation appears to depend on the condition: LH, RH, or bimanual hand finger tapping movements. Our results are in accord with previous studies. Both SMA and LPMC are known to be involved in movement selection and execution of movements, especially PMC which is thought to be involved in the execution of triggered movements and the transformation of external stimuli to motor planning (Lutz et al., 2000; Schubotz et al., 2001). During unimanual tasks, we found that SMA positively influences LM1 with 75 and 42% modulation for LH and RH finger movements respectively. Previously, SMA was found to be involved in various unimanual tasks. A transcranial magnetic stimulation (TMS) study by Arai et al. (2012) found excitatory influence from SMA to M1, which was in accordance with an fMRI study by Pool et al. (2013). On the other hand, SMA is also involved in temporal organization of bimanual movements. We find strong modulation between SMA and LM1 during BH finger movement. Anatomical studies also show SMA is connected with various motor areas in opposite hemispheres confirming its role during bimanual tasks (Rouiller et al., 1994). A study involving patients with lesions affecting SMA demonstrated upper limb impairments during bimanual tasks (Serrien et al., 2001) and a study using non-human primates demonstrate the role of SMA and M1 during bimanual task execution (Donchin et al., 1998). These studies showed that SMA neurons fire significantly during bimanual tasks whereas firing pattern of neurons differs when compared between unimanual and bimanual movements.

We compared the connection strengths and directionality between SMA and LM1 for btRS and ME. We observed that this connection is suppressed during preparation to execute the task. Hence if planning, preparation and then execution of motor task are considered in the same domain, we find a closed-loop circuit between both of these areas where there is feed-forward influence from LM1 to SMA during preparation and feed-backward influence from SMA to LM1 during actual execution of task.

Previous studies compare the effective connectivity between MI and ME using other approaches like DCM and SEM and find similar results suggesting positive influence of task resulting in strong activation of M1 and a suppressive influence during MI but strong enough to keep M1 active to execute the task (Solodkin et al., 2004; Kasess et al., 2008). Further, strong influences of LPMC on LM1 were found in unilateral as well as BH finger movements whereas a weak and insignificant influence was observed during btRS. This result may have occurred due to the lack of actual movements during btRS, resulting in a suppression of activity in LM1 with a concomitant increase in activation for LPMC and SMA. Further, a large proportion of activation is found during kinesthetic imagery (KI) in LPMC. It was suggested that KI can be considered as a part of motor preparation system (Solodkin et al., 2004). We found networking during the btRS and ME tasks to be very similar with activations in the same cortical areas where roles of SMA and LM1 differ as one source and other target depending on whether its analyzed before or after the actual task is executed. This is consistent with the study by Solodkin and colleagues which suggests that changes in interrelationships among these areas result into different prospects of the same network (Solodkin et al., 2004).

In this study, we used the bivariate version of the Geweke's spectral decomposition-based GC (Geweke, 1982; Dhamala et al., 2008a) for the calculation of directed functional connectivity. In the directed transfer-function based formulation (Kuś et al., 2004), the bivariate methods are shown to have limitations for correctly resolving true directions. A complete set of recordings is required to resolve true patterns of interactions. Geweke's bivariate formulation has a limitation of distinguishing indirect causal influences from direct causal influences. The reduction in conditional GC (from node 1 to node 2 conditioned on node 3) compared to pairwise causality (from node 1 to node 2) (Dhamala et al., 2008a) can indicate a mediated causal influence (between 1 and 2 via 3). The sensorimotor regions we considered in this study are known to have direct anatomical connections with each other. Therefore, conditional causality was not used here. The main conclusions are based on the low-frequency GC spectra and the connectivity modulation by the motor tasks, for which the use of bivariate GC is sufficient. Although the understanding of the exact mechanism for slow (<0.1 Hz) oscillations is lacking, the oscillatory node and network activity as observed here from the fNIR measurements could be due to neural level excitability fluctuations (Elwell et al., 1999; Balduzzi et al., 2008; Tong and Frederick, 2010) and communication in cortical and subcortical networks (Buzsáki and Draguhn, 2004; Balduzzi et al., 2008; Keilholz et al., 2010). Excitability fluctuations allow for the flexibility in moment-to-moment perception, cognition, and motor behaviors (Arieli et al., 1996; Makeig et al., 2004; Palva and Palva, 2012). The neural mechanism into how slow oscillations are generated and associated across electrical, metabolic and hemodynamic processes still remains a topic of future investigations, which may require these processes to be monitored concurrently.

In conclusion, results of the present work show (i) power, coherence and GC spectra had peaks within the frequency band (0.01–0.04 Hz) during RS whereas the peaks shifted to a higher frequency range (0.04–0.08 Hz) during btRS and finger movement tasks, (ii) there was significant bidirectional connectivity between all the nodes during RS and unidirectional connectivity from the LM1 to SMA and LM1 to LPMC during btRS, and (iii) the connections from SMA to LM1 and from LPMC to LM1 were significantly modulated in LH, RH, and BH finger movements relative to btRS. These results are consistent with the other studies, which used fMRI and EEG techniques. This provides us confidence that NIRS can be effectively used for monitoring slow hemodynamic fluctuations and underlying brain functional connectivity during rest and task. These data serve as a foundation for studies to follow comparing the characteristics of motor and RS networks between healthy subjects and people with neurologic insult such as stroke. Analysis of brain network in patients with lesions may lead to an effective approach to determine the functional and structural damage to cortical network connections, which may better inform us as we develop clinical recovery pathway for these clients.

## Author contributions

Mukesh Dhamala, Andrew J. Butler, Daniel Drake, and Sahil Bajaj wrote manuscript. Sahil Bajaj, Daniel Drake and Mukesh Dhamala analyzed data. Mukesh Dhamala, Andrew J. Butler, Daniel Drake, and Sahil Bajaj provided concept/idea/research design and project management.

### Conflict of interest statement

The authors declare that the research was conducted in the absence of any commercial or financial relationships that could be construed as a potential conflict of interest.
